# Nonequilibrium Electrochemical Phase Maps: Beyond Butler–Volmer Kinetics

**DOI:** 10.1021/acs.jpclett.3c01992

**Published:** 2023-08-24

**Authors:** Rachel C. Kurchin, Dhairya Gandhi, Venkatasubramanian Viswanathan

**Affiliations:** † Carnegie Mellon University, 5000 Forbes Ave, Pittsburgh, Pennsylvania 15213, United States; ‡ JuliaHub, https://juliahub.com

## Abstract

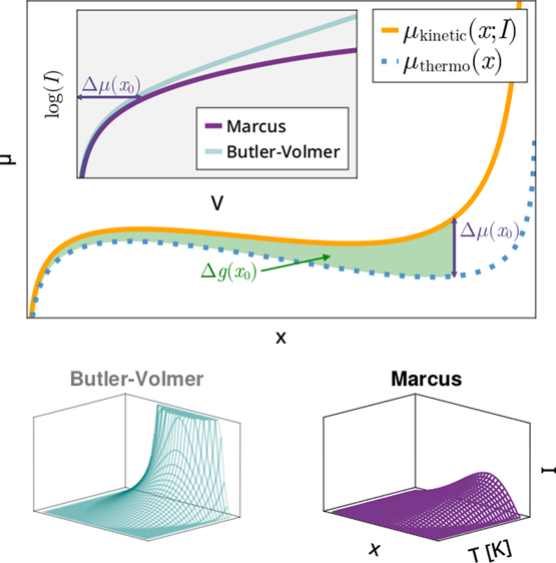

Accurate models of electrochemical kinetics at electrode–electrolyte interfaces are crucial to understanding the high-rate behavior of energy storage devices. Phase transformation of electrodes is typically treated under equilibrium thermodynamic conditions, while realistic operation is at finite rates. Analyzing phase transformations under nonequilibrium conditions requires integrating nonlinear electrochemical kinetic models with thermodynamic models. This had only previously been demonstrated for Butler–Volmer kinetics, where it can be done analytically. In this work, we develop a software package capable of the efficient numerical inversion of rate relationships for general kinetic models. We demonstrate building nonequilibrium phase maps, including for models such as Marcus–Hush–Chidsey that require computation of an integral, and also discuss the impact of a variety of assumptions and model parameters, particularly on high-rate phase behavior. Even for a fixed set of parameters, the magnitude of the critical current can vary by more than a factor of 2 among kinetic models.

Energy storage via batteries plays a vital role in the electrification transformation that is crucial to addressing climate change. Fast charging capabilities are important to meet consumer demand in passenger electric vehicles,^1^ and fast discharge will be pivotal to enable emerging applications such as electric aviation.^2,3^ Modeling the behavior of electrochemical systems at these high rates necessitates going beyond Butler–Volmer (BV) kinetics, as BV is a first-order approximation to the activation energy of a reaction valid only at small overpotentials. The larger overpotentials required for high-rate applications requires adoption of higher-order (and more physically interpretable^4^) models such as Marcus theory,^5^ Marcus–Hush–Chidsey (MHC) models,^6^ or our recently introduced modification, Marcus–Hush–Chidsey–Kurchin–Viswanathan (MHCKV), that incorporates electrode densities of state (DOS) explicitly.^7^ In addition, even at lower rates, a variety of systems have been shown to deviate from BV kinetics.^8−10^

Beyond deviation from BV, high rates can also impact phase behavior,^11−15^ causing deviation from thermodynamic phase diagrams. Previous work modeled this for the case of BV kinetics of intercalation into LiFePO_4_ (LFP) nanoparticles.^16^ Here, we show for the first time the capability to build nonequilibrium phase maps for any kinetic model with any underlying thermodynamic parameters. To do so, we introduce ElectrochemicalKinetics.jl (accessible from the Julia General registry, and also on GitHub at https://github.com/BattModels/ElectrochemicalKinetics.jl), a Julia language package that provides a common interface for a variety of rate models, including BV, Marcus, MHC, Zeng et al.’s asymptotic approximation to MHC,^17^ and our DOS-dependent model. Implementation of additional rate laws (such as asymmetric Marcus kinetics^18,19^) is straightforward. Crucially, ElectrochemicalKinetics.jl can not only evaluate them in the “forward” direction (computing a current for a given overpotential, *I*(η)) but also makes use of automatic differentiation (AD) to efficiently “invert” them, computing the overpotential required to drive a given current under a particular model, η(*I*). This “inverse” function is needed for building phase maps in the general case, where, unlike BV kinetics with symmetric electron transfer, there is no closed-form inverse.

Continuing with the example of Li intercalation into LFP, we consider the reaction:
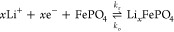
1where *x* represents a concentration of Li in iron phosphate (and 1 – *x* the concentration of “vacant” sites). As has been done previously,^16^ we model the thermodynamics via a regular solution model, with an interaction parameter Ω describing the energy of interaction between an intercalated Li and a vacant site. A smaller value of Ω will tend to promote homogeneous mixing at all *x*, while a larger value will promote phase separation between fully intercalated regions and those with no intercalants (in the LFP example, LiFePO_4_ and FePO_4_, respectively). The molar Gibbs free energy of mixing is then given by
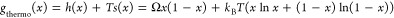
2

Then, by definition, the chemical potential is
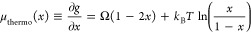
3

To incorporate the effects of a steady-state intercalation current *I*, we modify eq 3 by adding a term to account for the overpotential needed to drive that current:
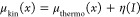
4Figure 1a shows a plot of both μ_thermo_(*x*) and μ_kin_(*x*) for the case of a Marcus model (rate relationship shown in inset, also see eqs 10 and 11 below) at an imposed current of 1/40 of the inversion current.

**Figure 1:**
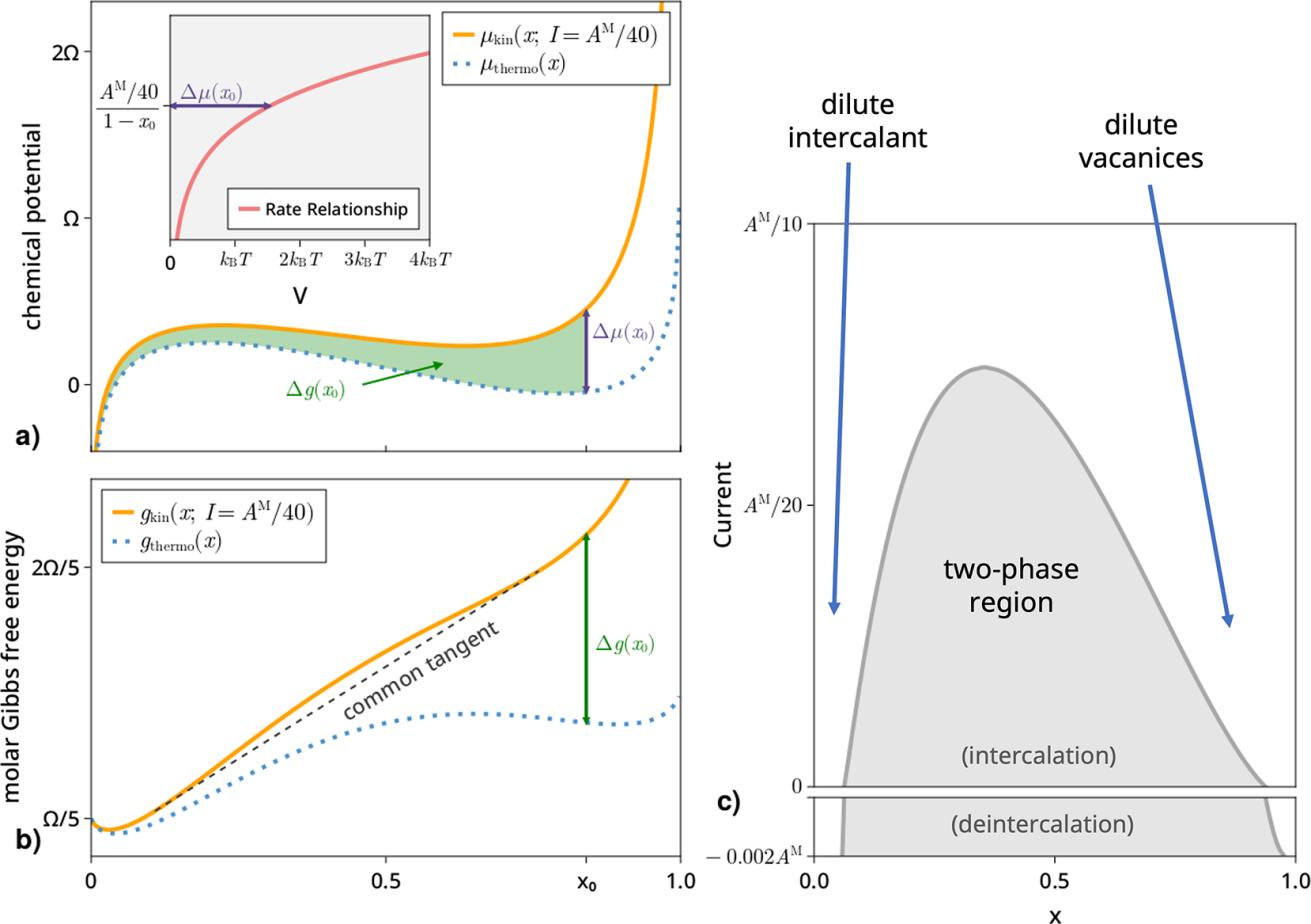
Nonequilibrium phase map construction. (a) Kinetic chemical potential is computed for a given rate model (inset) at a given current, here *A*^M^/40, where *A*^M^ is the prefactor (and maximum output value possible) for the Marcus model used. (b) Chemical potential is integrated to compute the associated molar Gibbs free energy. Phase boundaries at the imposed current are computed by using the common tangent condition. (c) This process is repeated at different currents to build up the phase map. Stripping is plotted as a negative current. For these plots, the interaction parameter Ω ≃ 3*k*_B_*T* and reorganization energy λ ≃ 10*k*_B_*T*.

The reaction rate (and thereby the current) has two parts: the intrinsic reaction rate of the process and available sites for the reaction.^20^ This necessitates a model for the activities of the intercalated Li atoms and the vacant sites; denote these as *a*_r_(*x*) and *a*_0_(*x*) (Bai et al.^16^ used *a*_0_(*x*) = *a*_r_(*x*) = 1 – *x*, based on an excluded volume argument; we discuss other possible choices for these functions and their implications below). The current is then given by
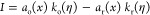
5where we have adopted the convention of oxidative current being positive, and the functions *k*_0_(η) and *k*_r_(η) represent the overpotential dependence of the rate constants in the oxidative and reductive directions under otherwise standard conditions (all nondimensional concentrations unity). In our analysis, we will assume a constant concentration of Li ions.

There are a variety of possible choices for the rate constant expressions *k*_0_(η) and *k*_r_(η). Prior work^16^ used Butler–Volmer (BV) kinetics:
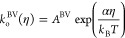
6
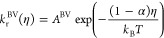
7where α is the electron transfer coefficient and *A*^BV^ is a prefactor with a weaker temperature dependence (we are working in a set of units where the charge on the electron is equal to unity).

BV kinetics is convenient because in the case of symmetric electron transfer (α = 0.5), the rate constant expression can be easily inverted. In particular, for symmetric activities (*a*_0_ = *a*_r_ = *a*), the expression for the current (eq 5) reduces to
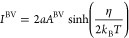
8and therefore, in this case,
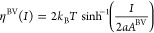
9(this statement is analogous to eq 9 in ref (16)).

A BV model will work well at modest overpotentials but can be expected to be a worse approximation at higher rates/overpotentials. In such cases, higher-order models are desirable. The simplest option among these is Marcus theory:^5^
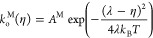
10
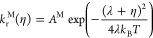
11where λ is the reorganization energy.

One could also include Chidsey’s modification to Marcus theory and use the so-called Marcus–Hush–Chidsey (MHC) model,^6^ which accounts for electron state occupation:
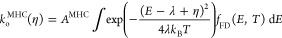
12
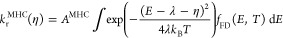
13where
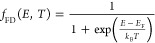
14is the Fermi–Dirac distribution (and *E*_F_ the Fermi energy).

Note that the need to compute an integral makes MHC kinetics substantially more computationally expensive. Zeng et al.^17^ introduced an asymptotic approximation to MHC kinetics that exhibits <10% error for most realistic parameter ranges:
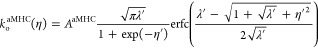
15
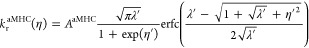
16where primed parameters have been nondimensionalized with respect to the thermal energy, i.e., λ′ ≡ λ/*k*_B_*T*.

The MHC model presumes a constant density of states (DOS). Our recent modification to it^7^ relaxes this assumption by explicitly integrating over the DOS  as well:
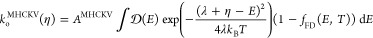
17
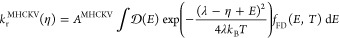
18

In ElectrochemicalKinetics, we make use of Julia’s multiple dispatch^21^ to use a common set of functions that apply to any rate model. One can use the rate_constant(eta, model,...) function with any KineticModel object (all the rate models described above are implemented as subtypes of the abstract KineticModel type). Similarly, we can obtain numerical inverses for any rate model (i.e., compute η(*I*)) via the overpotential(I, model;...) function, which uses automatic differentiation to do this via gradient-based optimization (see the Supporting Information for more details on this).

Continuing the analysis of the steady-state case introduced in eq 4, since it still must be true that μ(*x*) = ^δ*g*^/_δ*x*_, we then also have that
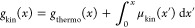
19With this expression and μ_kin_(*x*) (eq 4) in hand, we can use the usual common tangent construction (see Figure 1b) to find the phase boundaries as a function of the imposed current. At a given current, the phase boundaries can be computed with the function find_phase_boundaries(I, model,...), and a full phase map can be constructed using phase_diagram(model) (see the Supporting Information for more implementation details). Figure 1c shows the resulting nonequilibrium phase map with intercalation currents plotted as positive and deintercalation as negative.

Note that our analysis here presumes that intercalation is kinetically limited, as opposed to transport-limited. That is, we effectively consider the case of a uniform concentration. In future work, we plan to couple the software to a transport model such as the phase field model adopted in ref (16) in order to incorporate transport limitations and the effects of concentration gradients.

For more details on implementation and benchmarking, see the Supporting Information. Here, we remark that performant access to derivatives of rate models is crucial for efficient construction of these phase maps. In order to invert a generic rate model, a numerical solve is necessary (since no analytical inverse exists in general), and it is crucial that this calculation be efficient since the overpotential function is called many times (and is itself optimized over) in determining phase boundaries at a given current in order to build a phase stability map. This numerical solve is done via gradient-based optimization. These gradients can be accessed traditionally via finite differencing, but we find that for integral-based models, we obtain a substantial (∼50%) performance boost by using automatic differentiation. Currently, this is provided via Julia’s Zygote package,^22^ though we plan to transition to Enzyme^23,24^ once all necessary features are available (see more detailed discussion in the Supporting Information).

For the remainder of this work, we focus on the case of intercalation, where larger currents suppress phase separation. We now turn to the impact of the thermodynamic parameters (in the case of ideal mixing, the interaction parameter Ω) and kinetic model. We chose three temperatures and two values of the interaction parameter to demonstrate the qualitative range of observed behaviors. We parametrize the three non-integral-based models—Butler–Volmer (eqs 6 and 7), Marcus (eqs 10 and 11), and asymptotic MHC (eqs 15 and 16)—such that their low-overpotential behavior at room temperature is equivalent and then fix these parameters. Figure 2 illustrates the variation of the phase maps with model type, temperature, and interaction parameter. The Tafel plots for each model at each temperature are shown in the top row, and phase maps for two values of Ω are shown in the bottom two. (For an investigation of the impact of asymmetric electron transfer in a BV model, see the Supporting Information.)

**Figure 2:**
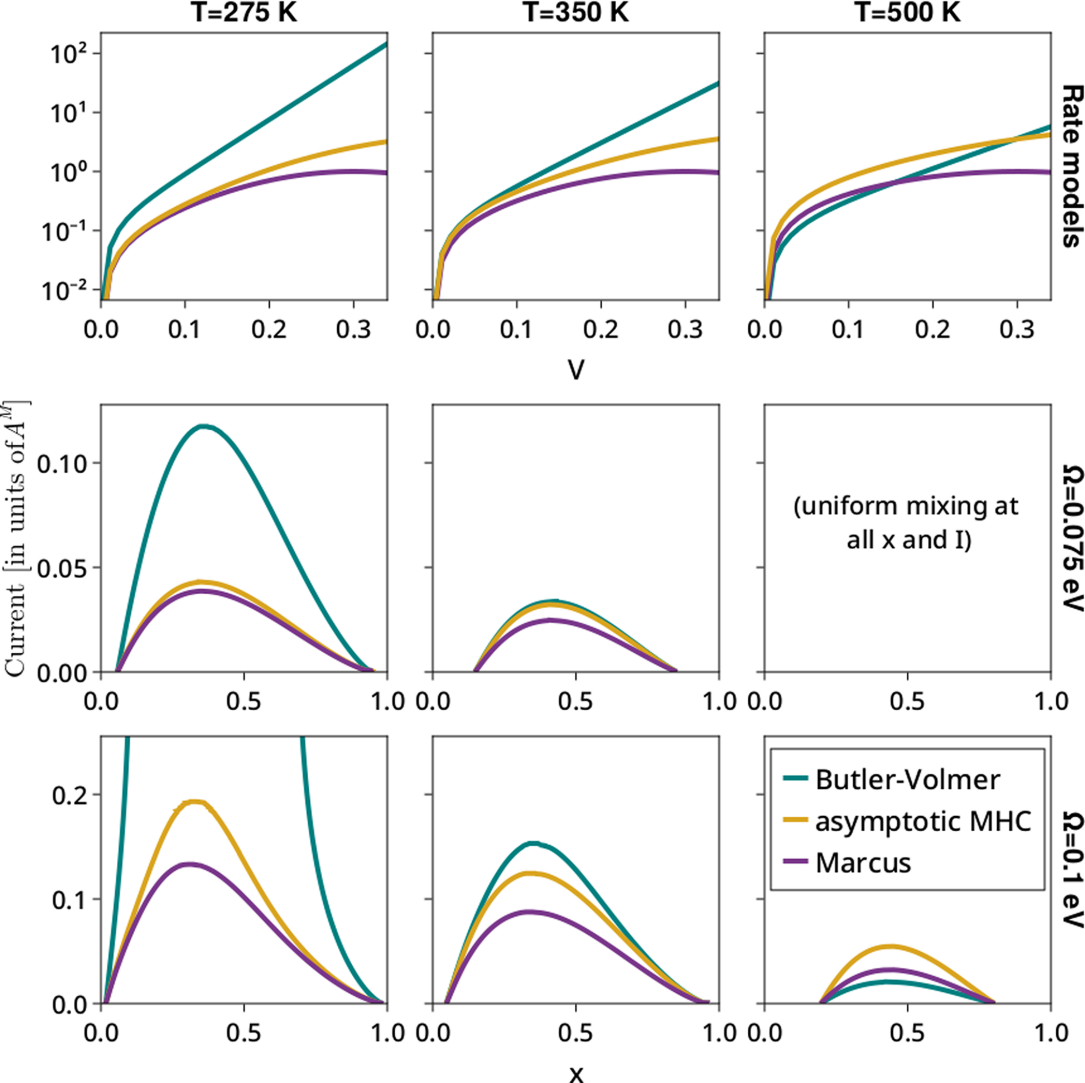
Phase maps for intercalation under three different rate models at three different temperatures (Tafel plots shown in the top row) and two different interaction parameters Ω (second and third rows). For the Marcus and MHC models, λ = 0.3 eV. Currents are nondimensionalized by the prefactor *A*^M^ of the Marcus model, and prefactors of all models are chosen such that low-overpotential Tafel plots coincide at 300 K.

Several interesting effects are apparent from this parameter sweep. First, there are substantial qualitative and quantitative differences between the phase maps under different kinetic models: most notably, at larger Ω and lower *T* for a Butler–Volmer model, there is no critical current beyond which phase separation stops, but rather there is a two-phase region at some intermediate range of compositions no matter how large the current; this is directly related to the fact that, unlike more physics-based models, a BV model can mathematically reach any output current with a large enough applied overpotential.

Second, we note the trends in the critical current with these parameters. It increases with increasing interaction parameter Ω and decreases with temperature, eventually yielding uniform mixing at all compositions, even with no current. These are both in fact thermodynamic effects, as the mixing model in eq 2 can easily be shown to have a critical temperature (above which there is uniform mixing) at Ω/2*k*_B_ (i.e., 435 K when Ω = 0.075 eV and 580 K when Ω = 0.1 eV) or, equivalently, a critical Ω (below which there is uniform mixing) of 2*k*_B_*T* (i.e., 0.047, 0.060, and 0.086 eV for temperatures of 275, 350, and 500 K, respectively). We also note that the trend of critical current with temperature is quite different for the three different models: critical current decreases substantially faster for BV models, such that the rank ordering between the three changes as *T* increases.

Figure 2 is, of course, simply a series of slices out of what could be considered a three-dimensional phase map, with critical current as a function of both *x* and *T*. Visualizations of these 3D phase maps can be found in the Supporting Information.

So far, we have considered only models that do not require evaluation of an integral to compute rate constants. ElectrochemicalKinetics.jl also supports integral-based models, such as the full (i.e., not asymptotically approximated) MHC model, and our recently introduced MHCKV variant involving the electrode DOS.

Phase maps for these models at three values of the reorganization energy λ are shown in Figure 3, along with the asymptotic approximation to MHC for comparison. Again, some interesting trends can be observed. First, we see a lowering of critical current with increasing reorganization energy, which is not surprising, as λ proximally sets the Tafel slope, and hence, a larger λ means a larger overpotential will be necessary to achieve any given current. In addition, we see that the agreement between the full solution of the MHC model and its asymptotic approximation improves with an increasing λ as well. This approximation being worse when λ is comparable to *k*_B_*T* is unsurprising, given that its construction involved taking the limits λ ≪ *k*_B_*T* and λ ≫ *k*_B_*T* and constructing an interpolation between these regimes.^17^

**Figure 3:**
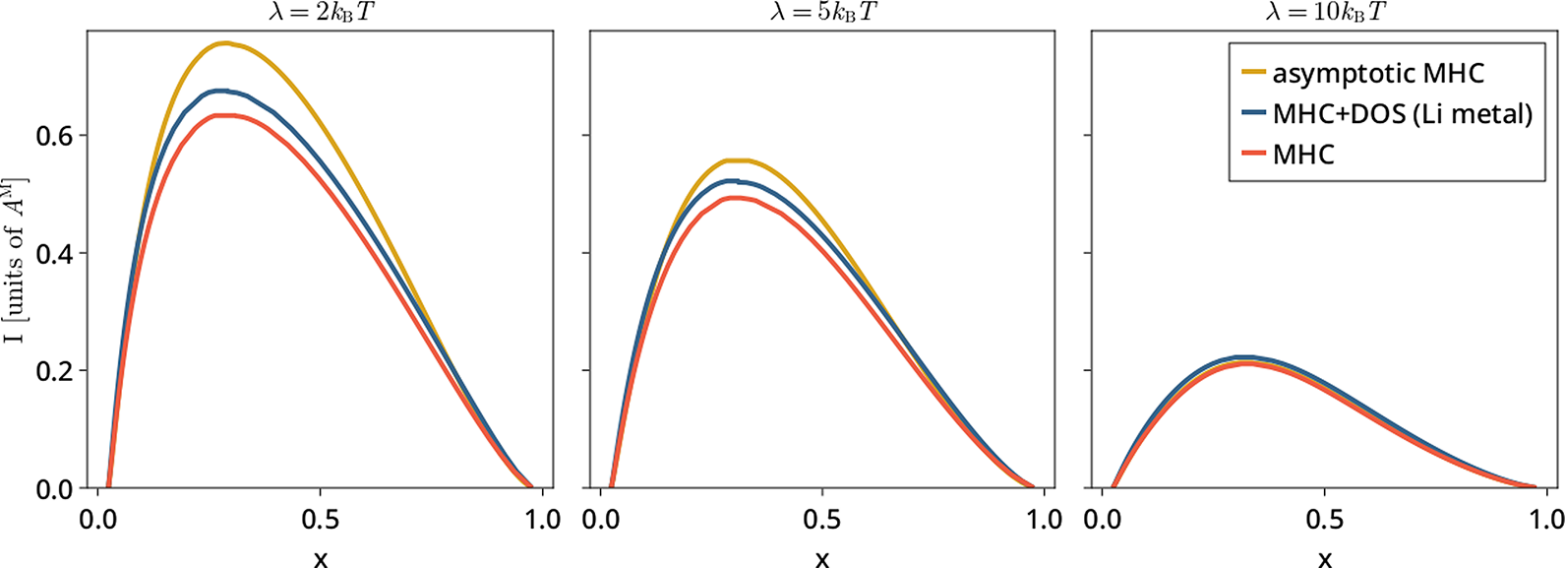
Phase maps for two integral-based MHC-based models at a variety of reorganization energies λ. Asymptotic approximation to MHC also shown for comparison. Currents are nondimensionalized as in Figure 2 by the prefactor *A*^M^ of the “equivalent” Marcus model.

Finally, we return to the matter of the model for the activity of the intercalated vs. nonintercalated sites. This may seem to be a minor point, but this assumption can have dramatic impacts on the resulting phase map. This is demonstrated in Figure 4, which shows a phase map for a Butler–Volmer model under three different activity models:

**Figure 4:**
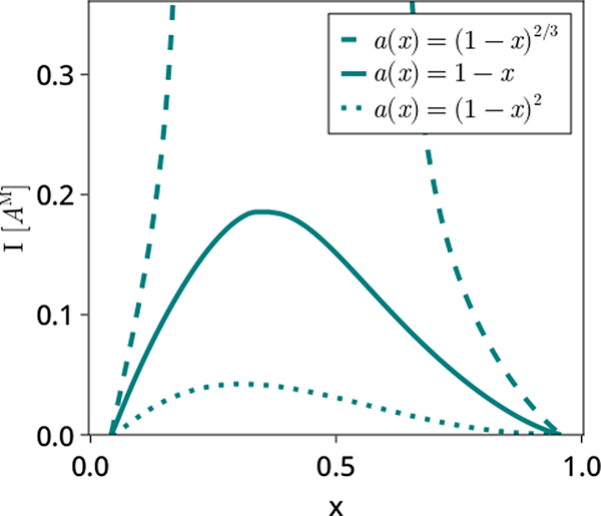
Phase maps for a Butler–Volmer model with three different models for activity: 1 – *x*, the one assumed by Bai et al.,^16^ (1 – *x*)^2^, corresponding to a two-site reaction scheme, and (1 – *x*)^2/3^, a surface reaction.

(i) 1 – *x*, the same one used for prior work and prior figures

(ii) (1 – *x*)^2/3^, representative of a surface-limited reaction

(iii) (1 – *x*)^2^, representative of a two-site reaction

It is clear from the figure that the nature of the activity has a substantial effect on the phase behavior. The activity model is passed in as a callable function, and so this approach can easily be extended to any function of concentration, such as a Debye-Hückel^25^ or TCPC^26^ model. Note that for simplicity, we have assumed there that *a*_o_ = *a*_r_, but this need not be the case.

To conclude, we have demonstrated the capability to construct nonequilibrium phase maps for any electrochemical rate relationship using the package ElectrochemicalKinetics.jl, which leverages the multiple dispatch feature of the Julia language to apply to any rate model as well as its full-featured automatic differentiation capabilities to improve performance, particularly on rate models that require computation of integrals.

The analysis enabled by this functionality compellingly demonstrates the importance of moving beyond simple Butler–Volmer models if accurate models of high-rate behavior are desired. In the future, we plan to integrate the package with battery modeling packages such as PyBAMM^27^ in order to couple it to charge transport (e.g., in a Doyle–Fuller–Newman model^28^) and assess the impact on device performance. It has already been used in studies examining electrochemical behavior of twisted graphene structures,^29^ where van Hove singularities create dramatic variations in the DOS, rendering a model like MHCKV necessary to capture device behavior.

All data shown in this work were generated using version 0.2.2 of ElectrochemicalKinetics.jl, and the code to generate figures can be found at https://github.com/BattModels/EK_paper.

## Supporting Information Available

More details about the software package ElectrochemicalKinetics.jl (including benchmarks), examples of 3D phase maps, phase maps for Butler–Volmer models with varying charge transfer coefficients, battery modeling checklist^30^ (PDF)
