# Supplementary material for: Nonequilibrium Electrochemical Phase Maps: Beyond Butler–Volmer Kinetics
Source: J Phys Chem Lett. 2023 Aug 24;14(35):7802–7. doi: 10.1021/acs.jpclett.3c01992 (PMC10494226; doi:10.1021/acs.jpclett.3c01992)
Supplement: Supplementary file 1 — jz3c01992_si_001.pdf [file jz3c01992_si_001.pdf]

Supporting Information for
Nonequilibrium Electrochemical Phase Maps: Beyond Butler-Volmer Kinetics

Rachel C. Kurchin,*,† Dhairya Gandhi,‡ and Venkatasubramanian Viswanathan*,†

†*Carnegie Mellon University*

‡*Julia Computing*

E-mail: rkurchin@cmu.edu; venkvis@cmu.edu

ElectrochemicalKinetics.jl

ElectrochemicalKinetics.jl can be found free and open-source on GitHub at `https://github.com/BattModels/ElectrochemicalKinetics.jl`, and is also a registered package in the Julia General registry. In this section, we summarize some of the package's main features, with an emphasis on performance-critical aspects. For more, we refer readers to the `README` file in the repository and a recent JuliaCon talk accessible at `https://www.youtube.com/watch?v=QUKBobLoykY`. All data shown in this work were generated using version 0.2.2 of the software.

API Overview

Computing rates and overpotentials

Virtually all functionality in the package is, directly or indirectly, built around the `rate_constant` function. It takes two required arguments, the overpotential and a `KineticModel` object (see

next subsection), and returns the rate constant at that overpotential within the given model. It takes optional arguments to modify the temperature, and to consider only oxidative or reductive rather than net rates.

The "inverse" of `rate_constant` is `overpotential`, which takes in a rate constant/current and a `KineticModel` object and returns the overpotential that would give rise to that current within the given model. It takes an optional argument for temperature, as well as several others that modify some of its internal behavior.

Because most rate models do not have analytical expressions for their inverses, `overpotential` performs a numerical solve to determine the overpotential. By default, automatic differentiation as implemented in Zygote.jl is used to accomplish this using a gradient-descent optimizer. This functionality can be turned off by passing `autodiff=false` as a keyword argument. The initial guess for the optimizer can also be supplied via the `guess` keyword.

We note also that some models are not one-to-one functions (e.g. a Marcus model that has an inverted region). This can lead to numerical instabilities in some cases, but if reasonable initial guesses are provided, usually does not lead to major issues.

Model types supported

`KineticModel` types are divided into `IntegralModel`s and `NonIntegralModel`s. `IntegralModel` types require computation of an integral for a single rate constant computation. These types include:

- `MarcusHushChidsey`, a standard Marcus-Hush-Chidsey (MHC) model parameterized by a prefactor and reorganization energy
- `MarcusHushChidseyDOS`, an implementation of our MHC variant variant incorporating electrode density of states (DOS), also includes a prefactor and reorganization energy, and also requires density of states information

`NonIntegralModel` types have a closed-form expression for rate constant as a function

of overpotential that does *not* require computation of an integral. These types include:

- `ButlerVolmer`, a standard Butler-Volmer model parameterized by a prefactor and a charge transfer coefficient
- `Marcus`, a Marcus model parameterized by a prefactor and a reorganization energy
- `AsymptoticMarcusHushChidsey`, an implementation of the asymptotic approximation to MHC and parameterized by a prefactor and a reorganization energy

See the main manuscript for the equations and relevant citations associated with all models.

The multiple dispatch functionality of Julia means that once we implement `rate_constant` for a given `KineticModel` type, all other functionality such as `overpotential`, as well as features discussed in subsequent sections, will "just work," as the generic implementations only rely on `rate_constant` being dispatched on these types. This also means that ElectrochemicalKinetics.jl is easy to extend with other rate models.

Phase map construction

Once the `overpotential` function works efficiently, implementation of Equations 1-5 is relatively straightforward. With these functions in hand, finding phase boundaries for a given value of the current just requires identifying the pair of points x that satisfy the common tangent condition, which states that states that compositions x_1 and x_2 are phase boundaries if

$$\frac{g_{\mathrm{kin}}(x_2) - g_{\mathrm{kin}}(x_1)}{x_2 - x_1} = \mu_{\mathrm{kin}}(x_1) = \mu_{\mathrm{kin}}(x_2) \tag{1}$$

For a given current, finding points that meet this condition can be done with off-the-shelf optimization tools. Construction of a phase map then requires finding these points for a range of current values. Since an optimization problem has to be solved for each current, we speed this up by utilizing the phase boundaries at a given current to inform the starting

guess for the optimizer at the next higher value of the current, which cuts down the number of iterations substantially. All this is automated using the `phase_diagram` function.

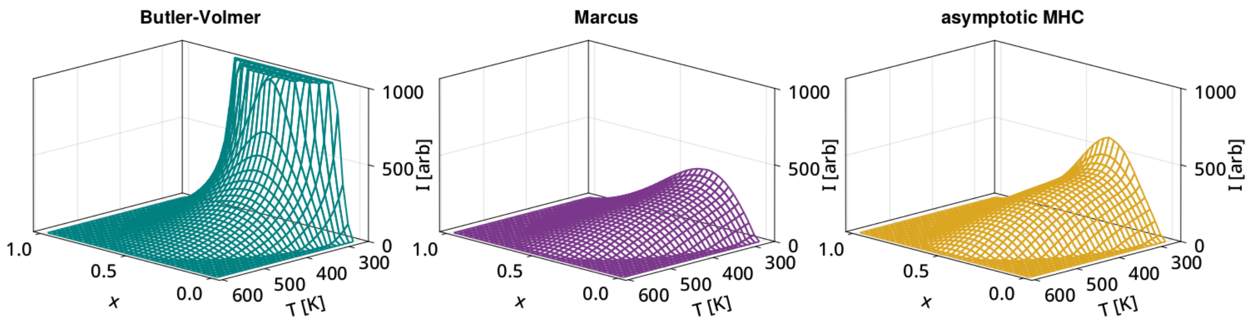

Figure 1: Three-dimensional phase maps for Butler-Volmer, Marcus, and asymptotic MHC models parameterized to have overlapping Tafel plots at low overpotential at 300K.

Figure 1 shows 3D phase maps (as a function of both composition and temperature) for the three non-integral models. The "floor" of these plots is the usual thermodynamic phase diagram in x and T.

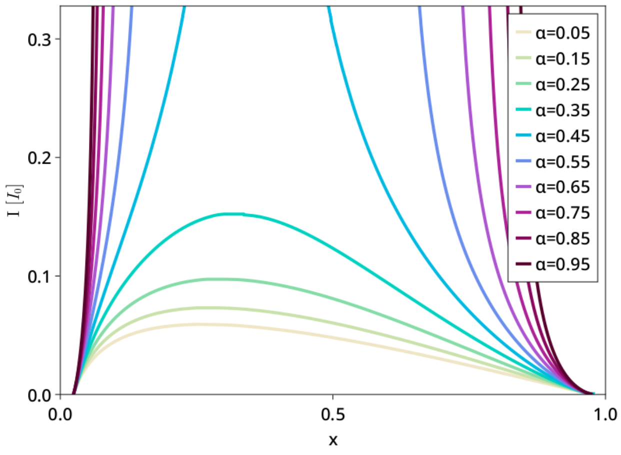

Figure 2: Phase maps at 300K for a Butler-Volmer model with a variety of charge transfer coefficients.

Figure 2 shows another parameter sweep not included in the main text: namely, the charge transfer coefficient α in a Butler-Volmer model. It has a qualitatively similar effect to temperature, but the actual composition at which the critical current occurs also shifts.

Other functionality

The package also has a variety of other functions for plotting/comparing Tafel plots of different rate models, fitting parameters of those models, as well as accounting for the effects of quantum capacitance, important for electrocatalysis at interfaces with 2D materials such as twisted bilayer graphene.

Performance

Here we present timing results obtained via the BenchmarkTools.jl package. We focus on the `overpotential` function, as it represents the core novelty of the package, and also is a "workhorse" function, being called many times and optimized over in construction of phase maps. The table below compares timings of calling this function with and without automatic differentiation on a range of kinetic models.

All tests were done using 64-bit arithmetic on a 3.2 GHz Intel Xeon W processor using Julia 1.9.2 and ElectrochemicalKinetics 0.2.2. The full benchmark suite can be found in the GitHub repository for the package at: `https://github.com/BattModels/ElectrochemicalKinetics.jl/tree/main/benchmark`.

Some timing distributions have very fat tails on the long end, so we include median, mean, and standard deviation (σ) values. Numbers of tests varied but were always at least 40.

Table 1

Model	with AD					without AD				
	median	mean	σ	memory	allocs	median	mean	σ	memory	allocs
BV	80 μs	95 μs	264 μs	129 KiB	1k	57 μs	64 μs	196 μs	48 KiB	937
Marcus	114 μs	136 μs	326 μs	183 KiB	1.5k	82 μs	92 μs	237 μs	70 KiB	1.4k
aMHC	107 μs	128 μs	317 μs	174 KiB	1.5k	80 μs	91 μs	244 μs	72 KiB	1.4k
MHCKV	119 ms	119 ms	1.6 ms	42 MiB	645k	237 ms	237 ms	2.7 ms	82 MiB	1.3M

One key takeaway is that AD does not provide a performance advantage for non-integral models at present – this is likely because we are currently using an AD system (Zygote.jl)

designed for machine learning applications, and hence optimized for differentiating large vectors and matrices. We are working on converting the package to work with Enzyme.jl, a newer AD system better suited to scientific computing, which often involves many scalar functions. Preliminary results indicate that Enzyme provides approximately a 10× improvement in both runtime and memory allocation, but it doesn't yet fully support all functionality that we require in ElectrochemicalKinetics, hence the main branch of the package still uses Zygote.

AD does both speed up and reduce allocation count for integral-based models, which are about three orders of magnitude slower (and two orders of magnitude more memory-hungry) to begin with.

Battery Modeling Checklist

Manuscript Title:	Nonequilibrium Electrochemical Phase Maps: Beyond Butler-Volmer Kinetics	
Submitting Author*:	Rachel Kurchin	

#	Question	Y/N/NA†
1	Have you provided all assumptions, theory, governing equations, initial and boundary conditions, material properties, e.g., open circuit potential (with appropriate precision and literature sources), constant states, e.g., temperature, etc.?<br>**Remarks:**	Y
2	If the calculations have a probabilistic component (e.g. Monte Carlo, initial configuration in Molecular Dynamics, etc.), did you provide statistics (mean, standard deviation, confidence interval, etc.) from multiple (≥ 3) runs of a representative case?<br>**Remarks:** Calculations do not have a probabilistic component.	NA
3	If data-driven calculations are performed (e.g. Machine Learning), did you specify dataset origin, the rationale behind choosing it, what all information does it contain and the specific portion of it being utilized? Have you described the thought process for choosing a specific modeling paradigm?<br>**Remarks:** No data-driven calculations are performed.	NA
4	Have you discussed all sources of potential uncertainty, variability, and errors in the modeling results and their impact on quantitative results and qualitative trends? Have you discussed the sensitivity of modeling (and numerical) inputs such as material properties, time step, domain size, neural network architecture, etc. where they are variable or uncertain?<br>**Remarks:**	Y
5	Have you sufficiently discussed new or not widely familiar terminology and descriptors for clarity? Did you use these terms in their appropriate context to avoid misinterpretation? Enumerate these terms in the 'Remarks'.<br>**Remarks:**	Y

* I verify that this form is completed accurately in agreement with all co-authors, to the best of my knowledge.

† Y ≡ the question is answered completely. Discuss any N or NA response in 'Remarks'.
